# Incidence, characteristics, and clinical impact of serious adverse events in patients with breast cancer receiving antineoplastic treatment in the ambulatory setting

**DOI:** 10.1002/prp2.70020

**Published:** 2024-10-28

**Authors:** Zahieh AbuAloush, Wedad Awad, Ola Mashni, Farah Shkakhwa, Ayman Al‐Faris, Maryam Al‐Omari, Sara Nabulsi, Lama Nazer

**Affiliations:** ^1^ Department of Pharmacy King Hussein Cancer Center Amman Jordan; ^2^ Department of Nursing King Hussein Cancer Center Amman Jordan; ^3^ University of Zurich Zurich Switzerland

**Keywords:** antineoplastic agents, breast neoplasms, complications, drug‐related side effects and adverse reactions, emergency treatment

## Abstract

Patients with breast cancer experience various types of adverse events (AEs) during their treatment journey. We aimed to evaluate the incidence, characteristics, and impact of serious AEs in breast cancer patients receiving antineoplastic treatment in the ambulatory setting. A 4‐month prospective observational study that included patients with breast cancer treated in the chemotherapy infusion clinics. Patients were assessed for serious AEs, defined as any AE that resulted in a visit to the emergency department (ED) with or without hospital admission, or required any clinical intervention, which were considered as the addition of supportive medications or modifications to the treatment protocol. Characteristics of the patients and antineoplastic regimens as well as the type of AEs were recorded. During the study period, 1168 patients received 2547 cycles. The mean age was 50 ± 11.6 (SD) years and patients had received a median (IQR) of 3 (1–5) treatment cycles prior to enrollment. Among the study cohort, 465 patients(40%) developed at least one serious AE. A total of 660 (26%) cycles were associated with 757 AEs, which required ED visits, addition of supportive medications, and modifications to the treatment protocol in 58%, 29%, and 17% of the cycles, respectively. Most common AEs were musculoskeletal (*n* = 132, 17%) and gastrointestinal (*n* = 125, 16.5%). Taxane‐based regimens were associated with the most AEs (*n* = 286, 38%). In a cohort of patients with breast cancer treated in the ambulatory setting, 4 out of 10 patients developed at least one serious AE during the study period. Future research should identify measures to reduce the incidence and severity of such complications.

**Abbreviations**
AEs: adverse events
ANC: absolute neutrophils count
BMI: body mass index
ED: emergency department
KHCC: King Hussein cancer center

## 1 INTRODUCTION

Breast cancer is the most commonly diagnosed malignancy worldwide.^1^ Treatment includes surgery, radiotherapy, as well as various systematic antineoplastic agents.^2^ While these treatment modalities are effective, patients may experience a range of adverse events (AEs) that may necessitate unscheduled medical care during their treatment journey. These AEs may be related to the treatment received, the malignancy itself, or the underlying comorbidities.

Despite the high prevalence of breast cancer, there are limited real‐world studies that evaluated AEs encountered during the treatment journey. Most of the studies have focused on a certain type of AEs (e.g., nausea/vomiting, visits to the emergency department), a specific patient population (e.g., older women, metastatic disease), or a specific treatment regimen (e.g., targeted therapies), and most have been limited by their relatively small sample sizes.^3^, ^4^, ^5^, ^6^, ^7^

Therefore, we conducted this study to provide a real‐world reflection of serious AEs experienced by patients with breast cancer during their treatment journey. Since antineoplastic therapy is the most common treatment in this patient population and is mostly administered in the ambulatory setting, we aimed to evaluate the incidence, characteristics, and clinical impact of serious AEs experienced by patients with breast cancer receiving antineoplastic treatment in the ambulatory setting.

## 2 MATERIALS AND METHODS

This was a prospective observational study conducted in the chemotherapy infusion clinics at King Hussein Cancer Center (KHCC), between September and December 2021. KHCC is a 350‐bed comprehensive cancer teaching hospital located in Amman, Jordan, which provides comprehensive care and treatment to adult and pediatric patients with various types of solid and hematological malignancies. There are two adult chemotherapy infusion clinics at KHCC, which serve over 4000 patients per month, 6 days a week.

The study included patients with breast cancer who presented to the chemotherapy infusion units to receive parenteral antineoplastic treatment. Eligible patients were those who had received at least one cycle of their treatment protocol. Antineoplastic agents referred to chemotherapy, as well as biological, and hormonal treatments. Patients who came to the clinic to receive parenteral supportive therapy such as bisphosphonates and iron therapy, and those who came to receive oral chemotherapy were excluded.

During the visit, patients were assessed for any serious AEs they may have developed since their most recent antineoplastic treatment cycle. Serious AEs were defined as any AE that resulted in a visit to the emergency department (ED) with or without hospital admission or required a clinical intervention. Clinical interventions were defined as the need for addition of new supportive medications that were not initially included in the treatment protocol or changes to the treatment protocol (i.e., chemotherapy dose reduction or chemotherapy dose delay).^8^ Upon presentation to the infusion clinic, the clinical pharmacist or nurse asked patients if they had any visits to the ED or addition of new medications since the last treatment cycle. In addition, the chemotherapy protocol and clinical notes in the patient's medical records were reviewed to determine if the patient had any complications that met the criteria of serious AEs.

If the patient had developed more than one AE (e.g., diarrhea and renal failure), the complication was classified based on what was considered as the major adverse event that contributed to the ED visit, addition of medications, or changes to the treatment protocol. If the AE resulted in ED visit and additional supportive medications (e.g., ED visit for diarrhea and prescribing antidiarrheal medications), the complication was considered as one that required a visit to the ED. Adverse events that were recorded in the patients' medical record but were not associated with any of the consequences outlined earlier were excluded.

Using the electronic medical records, we extracted patient demographics, type and cycle of antineoplastic treatment, and type of serious AEs experienced since the administration of the last cycle of antineoplastic treatment. AEs were classified based on the system involved to cardiovascular, dermatological, endocrine/metabolic, gastrointestinal, genitourinary, hematological, hepatic, infectious, musculoskeletal, neurological, ophthalmic, otic, dental, renal, and respiratory. We used the Naranjo scale to assess the likelihood of each AE being related to the antineoplastic treatment; events were classified as definite, probable, possible, and doubtful.^9^

### 2.1 Statistical analysis

Continuous patient data were presented as mean and standard deviation (SD) for parametric and median with inter‐quartile range (IQR) if non‐parametric. Categorical data were presented as counts and percentages. All statistical analyses were completed using R version 4.0.5 (R Core Team, Vienna, Austria).

## 3 RESULTS

During the 4‐month study period, 1168 patients were included who received a total of 2547 treatment cycles. The mean age was 50 ± 11.6 (SD) and all patients were females who had received a median (IQR) of 3 (1–5) treatment cycles prior to enrollment in the study. Most of the patients were non‐smokers (*n* = 934, 80%), over half of the patients had no underlying comorbidities (*n* = 682, 58%), and 518 (44%) had metastatic disease. Table 1 outlines the characteristics for patients included in the study.

**TABLE 1:** Baseline characteristics of patients.

Variables		Total Patients (*n* = 1168)	Occurrence of adverse events?	
			No *n* = 703	Yes *n* = 465
Age (years) (median, IQR)		50 (15)	47 (14)	50 (17)
BMI (Median, IQR)		29 (8)	29 (8)	30 (9)
Obese (BMI ≥30) *N* (%)		539 (46%)	304 (43%)	235 (50%)
Smoker *N* (%)		234 (20%)	131 (19%)	103 (22%)
Metastatic disease *N* (%)		518 (44%)	319 (45%)	199 (42%)
Comorbidities *N* (%)	Zero	682 (58%)	435 (62%)	247 (53%)
	One	290 (25%)	171 (24%)	119 (26%)
	Two	146 (12%)	68 (10%)	78 (17%)
	Three	42 (4%)	22 (3%)	20 (4%)
	Four or above	8 (0.7%)	7 (1.0%)	1 (0.2%)
Peri Surgery period *N* (%)		520 (45%)	284 (40%)	236 (51%)
Number of cycles received at enrollment Median (IQR)		3 (4)	3 (4)	2 (3)
Antineoplastic Regimen *N* (%)	Single	747 (64%)	501 (71)	246 (53%)
	Combination	421 (36%)	202 (29%)	219 (47%)
Regimen Received *N* (%)	Anthracycline based	260 (22%)	120 (17%)	140 (30%)
	Biological based	209 (18%)	155 (22%)	54 (12%)
	Endocrine based	332 (28%)	269 (38%)	63 (14%)
	Taxanes based	308 (26%)	132 (19%)	176 (38%)
	Platinum based	11 (1%)	5 (1%)	6 (1%)
	Others	48 (4%)	22 (3%)	26 (6%)

Among the included patients, 465 (40%) experienced at least one serious AE during their assessed treatment cycles. Table 1 outlines the characteristics of patients who developed and those who did not develop serious AEs during their treatment journey. Among the evaluated cycles, 660 (26%) were associated with 757 serious AEs. The majority of the AEs required visits to the ED (*n* = 436, 58%). Most of the ED visits were for respiratory complications (*n* = 89, 20%) followed by gastrointestinal complications (*n* = 81, 19%) and pain (*n* = 73, 17%). Among the ED visits, 43 (10%) required hospital admission. Febrile neutropenia was the most common cause of admission (*n* = 19, 44%) and the mean length of hospital stay was 4 ± 1.7 (SD) days.

Addition of supportive medications was required for the management of 221 (29%) AEs, of which analgesics and antidiarrheal medications were the most commonly added medications for the management of 51 (23%) and 16 (7%) AEs, respectively. The remaining AEs (*n* = 100, 13%) included those that required adjustment to the treatment regimen due to laboratory findings, such as severe neutropenia, renal failure, or liver failure. In addition, 18 (4%) of the AEs that required ED visits and 12 (5%) that required the addition of supportive medications were associated with adjustment to the treatment protocol in the subsequent cycles.

**FIGURE 1:**
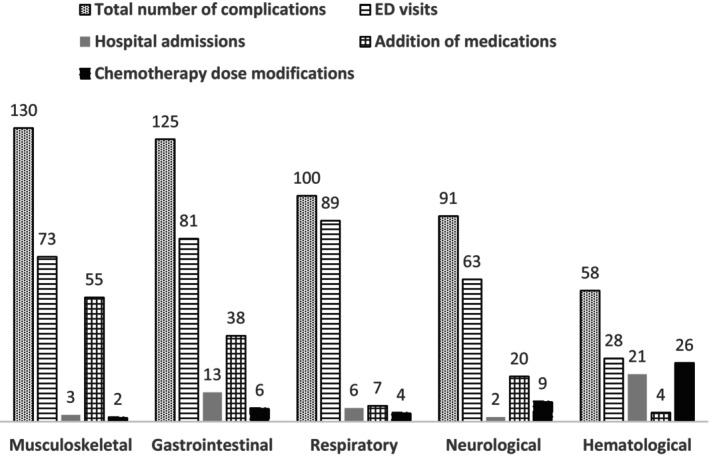
Characteristics of the top 5 adverse events during the study period.

The most common types of serious AEs were musculoskeletal reported in 130 (17%) cycles, followed by gastrointestinal in 125 (16%) cycles, respiratory in 100 (13%) cycles, and neurological in 91 (12%) cycles. Figure 1 demonstrates the characteristics of the most common types of AEs and the consequences associated with each type.

Serious AEs were higher in patients with non‐metastatic disease (*n* = 266, 57%) compared to patients with metastatic disease (*n* = 199, 43%). When comparing the type of serious AEs, the most common in patients with metastatic breast cancer were musculoskeletal (*n* = 68, 34%), neurological (*n* = 56, 28%), and respiratory (*n* = 51, 26%), while in patients with non‐metastatic disease, the most common serious AEs were gastrointestinal (*n* = 100, 38%), neurological (*n* = 63, 24%), and musculoskeletal (*n* = 63, 24%).

The majority of serious AEs were associated with taxane‐based regimens (*n* = 286, 38%) and anthracyclines (*n* = 175, 23%). The most common serious AEs associated with taxanes‐based regimens were gastrointestinal (*n* = 71, 25%), musculoskeletal (*n* = 59, 21%), and neurology (*n* = 57, 20%), while the most common reported with anthracyclines were gastrointestinal (*n* = 56, 32%), respiratory (*n* = 32, 18%), and hematological (*n* = 27, 15%).

According to the Naranjo scale, the causal relationship between the AEs and the antineoplastic treatment was definite in 6 (1%), probable in 389 (51%), possible in 319 (42%), and doubtful in 43 (6%) of the AEs.

## 4 DISCUSSION

In this study, we present an assessment of serious AEs experienced by patients with breast cancer receiving antineoplastic treatment in the ambulatory setting. We evaluated over 1100 patients with breast cancer treated in the infusion clinic over a 4‐month period; such patients had various stages of malignancy and received a wide range of antineoplastic agents. In this large cohort of patients who were relatively young (median age 50 years) and mostly healthy (58% with no comorbidities), 4 out of 10 patients experienced at least one AE that required additional clinical management. Although the incidence of serious AEs seems relatively high, this could be attributed to the prospective study design which involved close assessment of any AEs during the study period. The assessment of AEs in real‐world practice, rather than in controlled clinical studies, may have also contributed to such findings.

According to the Naranjo scale, most of the adverse events were considered as definitely, probably, or possibly associated with the administered antineoplastic agents. This study is unique in that it provides a full‐scope assessment of serious adverse AEs encountered during the treatment journey of patients with breast cancer. The findings provide insight to clinicians as well as administrators regarding the experience of a cohort of patients that is increasing globally.

Patients with cancer may present to the ED for various indications. In our study, over half of the adverse events necessitated visits to the ED. Such visits would add to the resources necessary for the management of patients with breast cancer, as well as impact the quality of life of patients and their families. In a large study that analyzed the Nationwide Emergency Department Sample from the US between 2006 and 2015, among 1.3 billion ED visits, 1.5 million were associated with complications from cancer systemic therapy and radiotherapy.^8^ In addition, the annual rate of increase in cancer treatment‐related ED visits was of 10.8%, compared to that reported for all ED visits, which was 2%.^10^

Few studies evaluated ED visits in patients with breast cancer. In a study that included 102 women with early‐stage breast cancer undergoing curative treatment with adjuvant and neoadjuvant chemotherapy, 39 (38%) patients had a total of 63 ED visits.^11^ The most common reasons for ED visits were non‐neutropenic fever (27%), neutropenic fever (24%), and pain (14%). In another study that included 149 breast cancer patients who completed at least one cycle of curative chemotherapy, 53% of the patients required at least one ED visit and 13% required hospital admission.^12^ The most common causes of ED visits were non‐neutropenic fever (23%), pain (13%), and febrile neutropenia (9%). Similar findings were reported by a larger study that included over 8000 patients with early‐stage breast cancer in which 43% of the patients had least one ED visit and hospitalization, which was higher than that for non‐cancer controls (9%).^13^ Though our study, as well as others, did not evaluate the preventability of the ED visits, the findings represent a potential opportunity for quality improvement that should be addressed in future research.

Patients with cancer may require the addition of supportive medications for the management of severe treatment‐related complications, such as nausea and vomiting, diarrhea, and pain. Though supportive treatments are included in many of the treatment protocols that are associated with high incidence of such adverse events, some patients may require further treatment beyond what is typically prescribed. In our study, about one‐fourth of the cycles required additional supportive therapies. In a study by Friese et al. that evaluated treatment‐associated toxicities reported by women with early‐stage invasive breast cancer (*n* = 1945), 175 (9%) patients reported unscheduled visits to the clinic for toxicity management.^14^ This represents another important area that requires further research to identify predictors for the need of additional supportive therapy and develop strategies to proactively address such complications.

The importance of maintaining chemotherapy dose and dose intensity in the treatment of breast cancer is highly emphasized in the literature.^15^, ^16^ Though adjustment to the treatment protocol carries with it the risk of suboptimal outcomes, especially in curable diseases, it is necessary in cases where the patients develop severe adverse events during the treatment journey. Adjustments to the chemotherapy dose intensity in patients with breast cancer ranged from 24% to 30%.^17^, ^18^ The findings reported in our study are similar to those reported in other studies, with the proportion approaching 20% for the adjustments associated with laboratory changes as well as those associated with ED visits or necessitating the addition of supportive medications. Identifying strategies to prevent or reduce the severity of AEs associated with antineoplastic treatment could facilitate the continuation of the treatment, as planned, to achieve optimal outcomes.

Our study has several limitations, which we would like to highlight. First is related to the time frame of the study which represents a relatively short duration of a treatment journey that generally extends for more than a year and may include more than one type of antineoplastic treatment. In addition, we did not evaluate the impact of AEs on the prognosis and outcomes of patients. We also did not evaluate the causes of AEs or predictors, which would help in identifying proactive measures for patients considered at high risk for such AEs. Nevertheless, this study sheds light on an important part of the treatment journey of patients with breast cancer and presents the incidence and characteristics of serious AEs that may impact patient outcomes and quality of life, as well as resource utilization.

In a cohort of patients with breast cancer treated in the ambulatory setting, 4 out of 10 patients developed at least one serious AE during their treatment journey. The most common types of reported AEs were musculoskeletal and gastrointestinal, and most complications required ED visits. Future research should identify measures to reduce the incidence and severity of such AEs.

## AUTHOR CONTRIBUTIONS

Zahieh AbuAloush, Lama Nazer, Ola Mashni and Wedad Awad contributed to the study conception and design. Material preparation and data collection were performed by Zahieh AbuAloush, Ola Mashni, Farah Shkakhwa, Ayman Al‐faris and Mariam Al‐Omari. Data analysis was performed by Zahieh AbuAloush and Sarah Nabulsi. The first draft of the manuscript was written by Zahieh AbuAloush, Lama Nazer, Sara Nabulsi and Wedad Awad and all authors reviewed all versions of the manuscript and approved the final manuscript.

## FUNDING INFORMATION

The study received an intramural grant from King Hussein Cancer Center to support a full‐time research assistant for the project.

## CONFLICT OF INTEREST STATEMENT

The authors declare that they have no competing interests.

## ETHICS STATEMENT

The study protocol was approved by the Institutional Review Board of King Hussein Cancer Center (# 20KHCC134) and was granted a waiver of informed consent, given the retrospective nature of the study. The data were coded and anonymized before analysis. The study followed the relevant guidelines and regulations of both good clinical practice guidelines and our Institutional Review Board.

## CONSENT FOR PUBLICATION

Not applicable.

## Data Availability

The datasets used and/or analyzed during the current study are available from the corresponding author on reasonable request.

## References

[1] Common cancer types . National Cancer Institute. Accessed 29 August, 2022. https://www.cancer.gov/types/common‐cancers.

[2] Nounou MI , ElAmrawy F , Ahmed N , Abdelraouf K , Goda S , Syed‐Sha‐Qhattal H . Breast cancer: conventional diagnosis and treatment modalities and recent patents and technologies. Breast cancer: basic and clinical. Research. 2015;9:9s2‐9s34. doi:10.4137/BCBCR.S29420 PMC4589089 26462242

[3] Hassett MJ , O'Malley AJ , Pakes JR , Newhouse JP , Earle CC . Frequency and cost of chemotherapy‐related serious adverse effects in a population sample of women with breast cancer. JNCI J Natl Cancer Inst. 2006;98(16):1108‐1117. doi:10.1093/jnci/djj305 16912263

[4] Garg P , Rana F , Gupta R , Buzaianu EM , Guthrie TH . Predictors of toxicity and toxicity profile of adjuvant chemotherapy in elderly breast cancer patients. Breast J. 2009;15(4):404‐408. doi:10.1111/j.1524-4741.2009.00745.x 19508671

[5] Delgado‐Ramos GM , Nasir SS , Wang J , Schwartzberg LS . Real‐world evaluation of effectiveness and tolerance of chemotherapy for early‐stage breast cancer in older women. Breast Cancer Res Treat. 2020;182(2):247‐258. doi:10.1007/s10549-020-05684-5 32447595

[6] Rashid N , Koh HA , Baca HC , et al. Clinical impact of chemotherapy‐related adverse events in patients with metastatic breast cancer in an integrated health care system. J Manag Care Spec Pharm. 2015;21(10):863‐871. doi:10.18553/jmcp.2015.21.10.863 26402387 PMC10398121

[7] Lima Cavalcanti ID , Silveira Cabral AG , Dos Santos RJ . Adverse reactions for the use of the monoclonal trastuzumab antibody in the treatment of patients with HER2 positive breast cancer. Ars Pharmaceutica (Internet). 2017;58(4):171‐174.

[8] Office for Human Research Protections (OHRP) (2021a) Unanticipated problems involving risks & adverse events guidance (2007), HHS.gov. Accessed 03 February 2024 https://www.hhs.gov/ohrp/regulations‐and‐policy/guidance/reviewing‐unanticipated‐problems/index.html.

[9] Evidencio (no date) Naranjo adverse drug reaction probability scale, Evidencio. Accessed: 10 August 2024 https://www.evidencio.com/models/show/661.

[10] Jairam V , Lee V , Park HS , et al. Treatment‐related complications of systemic therapy and radiotherapy. JAMA Oncol. 2019;5(7):1028‐1035. doi:10.1001/jamaoncol.2019.0086 30946433 PMC6583836

[11] Tang M , Horsley P , Lewis CR . Emergency department presentations in early stage breast cancer patients receiving adjuvant and neoadjuvant chemotherapy. Intern Med J. 2018;48(5):583‐587. doi:10.1111/imj.13785 29722200

[12] Pittman NM , Hopman WM , Mates M . Emergency room visits and hospital admission rates after curative chemotherapy for breast cancer. J Oncol Pract. 2015;11(2):120‐125. doi:10.1200/JOP.2014.000257 25585617

[13] Enright K , Grunfeld E , Yun L , et al. Population‐based assessment of emergency room visits and hospitalizations among women receiving adjuvant chemotherapy for early breast cancer. J Oncol Pract. 2015;11(2):126‐132. doi:10.1200/JOP.2014.001073 25604597

[14] Friese CR , Harrison JM , Janz NK , et al. Treatment‐associated toxicities reported by patients with early‐stage invasive breast cancer. Cancer. 2017;123(11):1925‐1934. doi:10.1002/cncr.30547 28117882 PMC5444953

[15] Qi W , Wang X , Gan L , Li Y , Li H , Cheng Q . The effect of reduced RDI of chemotherapy on the outcome of breast cancer patients. Sci Rep. 2020;10(1):13241. doi:10.1038/s41598-020-70187-8 32764734 PMC7413525

[16] Lyman GH . Impact of chemotherapy dose intensity on cancer patient outcomes. J Natl Compr Cancer Netw. 2009;7(1):99‐108. doi:10.6004/jnccn.2009.0009 19176210

[17] Weycker D , Barron R , Edelsberg J , Kartashov A , Lyman GH . Incidence of reduced chemotherapy relative dose intensity among women with early stage breast cancer in US clinical practice. Breast Cancer Res Treat. 2012;133(1):301‐310. doi:10.1007/s10549-011-1949-5 22270932

[18] Lyman GH , Dale DC , Crawford J . Incidence and predictors of low dose‐intensity in adjuvant breast cancer chemotherapy: a nationwide study of community practices. J Clin Oncol. 2003;21(24):4524‐4531. doi:10.1200/JCO.2003.05.002 14673039

